# Biological Effects and Biodistribution of Bufotenine on Mice

**DOI:** 10.1155/2018/1032638

**Published:** 2018-05-31

**Authors:** Hugo Vigerelli, Juliana Mozer Sciani, Maria Andrea Camarano Eula, Luciana Almeida Sato, Marta M. Antoniazzi, Carlos Jared, Daniel C. Pimenta

**Affiliations:** ^1^Laboratory of Biochemistry and Biophysics, Butantan Institute, SP, Brazil; ^2^Special Laboratory for Applied Toxinology, Butantan Institute, SP, Brazil; ^3^Laboratory of Cell Biology, Butantan Institute, SP, Brazil

## Abstract

Bufotenine is an alkaloid derived from serotonin, structurally similar to LSD and psilocin. This molecule is able to inhibit the rabies virus infection in* in vitro* and* in vivo* models, increasing the survival rate of infected animals. Being a very promising molecule for an incurable disease and because of the fact that there is no consensus regarding its neurological effects, this study aimed to evaluate chronic treatment of bufotenine on behavior, pathophysiology, and pharmacokinetics of mice. Animals were daily treated for 21 consecutive days with 0.63, 1.05, and 2.1 mg/animal/day bufotenine and evaluated by open field test and physiological parameters during all the experiment. After this period, organs were collected for histopathological and biodistribution analysis. Animals treated with bufotenine had mild behavioral alterations compared to the control group, being dose-response relationship. On the other hand, animals showed normal physiological functions and no histological alterations in the organs. With high doses, an inflammatory reaction was observed in the site of injection, but with no cellular damage. The alkaloid could be found in the heart and kidney with all doses and in the lungs and brain with higher doses. These results show that the effective dose, 0.63 mg/day, is safe to be administered in mice, since it did not cause significant effects on the animals' physiology and on the CNS. Higher doses were well tolerated, causing only mild behavioral effects. Thus, bufotenine might be a drug prototype for rabies treatment, an incurable disease.

## 1. Introduction

Bufotenine, a tryptamine alkaloid resulting from the methylation of serotonin, is a common metabolite spread throughout different living organisms, that can be found, for instance, in the skin secretion of many Brazilian toads of* Rhinella* genus [1] as well as in plants of* Leguminosae* family [2, 3]. Although bufotenine isolation was described in the 1920s [4], the literature still debates the possible hallucinogen effects of this molecule, due to its structural similarities to LSD and psilocin (Table 1). So far, no conclusive study has ever been conducted [5–11].

When performing biological-driven studies, our group found that bufotenine can inhibit rabies virus infection on mammalian cultured cells, in a dose- and time-dependent manner [12]. It also presents a synergic antiviral effect with a synthetic tetrapeptide derived from the natural antimicrobial peptide ocellatin-F1, which sequence is similar to that of the rabies virus glycoprotein region supposed to mediate the virus internalization [13, 14].

Moreover, bufotenine was able to prevent the development of* in vivo* rabies symptoms in 40% of intracerebral virus-infected mice, when administered daily, via subcutaneous route, versus 15% survival in the untreated group (manuscript submitted), without displaying disabling central nervous system symptoms. According to our data, bufotenine showed to act through an apparent competitive mechanism of action with the rabies virus for the cellular receptors/molecules responsible for its internalization. A direct cellular effect, involving cytoskeleton alterations, was also observed. Nevertheless, thorough characterization of this bioactive effect requires complementary studies in order to be fully elucidated.

The rabies virus infection model was chosen for it is an incurable zoonosis that kills more than 40 thousand people every year, mainly in Asia and Africa [15, 16], to which the only existing treatment is based on rabies vaccine and immune globulin treatment after exposure [17]. The speed of the onset of the treatment is crucial for the success of the therapy for the infected subjects. Other than this, there is no available treatment and the evolution of the disease culminates in the patient's death. A coma-induced treatment based on broad-spectrum antiviral drugs has been reported, named as Milwaukee protocol [18], but the literature on the subject is controversial and the medical authorities seek for the development of more specific drugs [19].

Taking into account the potential biotechnological and pharmacological uses of bufotenine (and synthetic analogues) and the fact that there is still debate whether this molecule causes any neurological effect, the aim of this study was to evaluate the chronic treatment of a daily bufotenine treatment on mice, analyzing possible behavioral, biological, and pathophysiological effects.

## 2. Material and Methods

### 2.1. Animals

Heterogenic Swiss mice (50% male and 50% female; 17 - 21 g) were housed, 5 per cage (separated by sex), at a room temperature of 22 ± 2°C and a 12 h : 12 h light/dark cycle. They had free access to food and water. All* in vivo* experiments were approved by the Ethic Committee on Animal Use of the Butantan Institute (CEUAIB), under protocol number CEUA 9532050216, which was in accordance with the rules issued by the National Council for Control of Animal Experimentation (CONCEA).

### 2.2. Reagents and Bufotenine

All reagents were of analytical grade and were purchased from Sigma Aldrich (USA), unless otherwise stated.

*Anadenanthera colubrina* seeds were obtained from the legitimate supplier Arbocenter Comércio de Sementes Ltda, Birigui, São Paulo (batch 0019), Brazil, and bufotenine was purified as previously described by [12].

### 2.3. *In Vivo* Experiments

For* in vivo* experiments, animals were grouped (10 animals each) according to the following treatments:

- Group 1, subcutaneous inoculation of NaCl 0.9% 250 *μ*l/animal/day (control group)
- Group 2, subcutaneous inoculation of bufotenine 0.63 mg in 250 *μ*l of NaCl 0.9%/animal/day (equivalent to 30 mg/kg initial dose)
- Group 3, subcutaneous inoculation of bufotenine 1.05 mg in 250 *μ*l of NaCl 0.9%/animal/day (equivalent to 50 mg/kg initial dose)
- Group 4, subcutaneous inoculation of bufotenine 2.10 mg in 250 *μ*l of NaCl 0.9%/animal/day (equivalent to 100 mg/kg initial dose).

The animals were treated once a day, for 21 days (WHO preconized rabies protocol [20]), and were observed daily, for two hours following bufotenine injection, in order to observe any possible sign or symptom of the daily exposure to this alkaloid. The animals were weighed on day 1 and then every 4 days until the end of the experiment.

The open field experiments were performed on days 1, 7, 14, and 21. After euthanasia, skin samples of the bufotenine inoculation sites were collected for histological analysis. Brain, liver, heart, kidney, lung, pancreas, and spleen were also collected for histological analysis and to evaluate the presence of bufotenine in these organs.

### 2.4. Open Field Test

This method allows the simultaneous assessment of the locomotor activity, the level of exploration, and the anxiety-related behaviors in rodents [21–23]. The open field consisted of a 40 cm diameter circular area, divided into 32 quadrants, surrounded by a 19 cm wall. Twenty minutes after treatment, each mouse was individually placed in the center of the arena (previously cleaned with ethanol 20%) and the number of crossed lines (i.e., the mice crossed one of the grid lines with all four paws), rearing (i.e., the mice stood on their hind legs), time to leave the center, and frequency of defecation and urination were manually recorded for 5 min.

### 2.5. Biodistribution of Bufotenine

Mice were anesthetized with carbon dioxide (CO_2_), and organs were immediately collected in 40 mM Tris buffer, containing 7 M urea, 2 M thiourea, 4% CHAPS, and 50 mM DTT and lysed by sonication for five cycles of 20 kHz each, for 30 s, on ice. Trichloroacetic acid (0.1 M) was added, followed by centrifugation at 12 000 x g for 15 min. The supernatant was collected, lyophilized, and homogenized in MeOH, for bufotenine extraction. Then, samples were analyzed by reversed-phase liquid chromatography coupled to mass spectrometry (LC-MS/MS).

For LC-MS/MS analyses, samples were injected into a C18 reversed-phase column (Supelco, 3 *μ*m, 100 Å, 50 mm × 2.1 mm) coupled to a Proeminence 20A binary HPLC (Shimadzu) and eluted with a 0–100% gradient of solvent B (90% acetonitrile/H_2_O with 0.1% formic acid) over 30 min, at a constant flow rate of 0.2 mL.min^−1^. MS spectra were acquired on a IT-ToF (Shimadzu Co, Japan), in which the spray voltage was kept at 4.5 KV, the capillary voltage at 1.76 KV, and interface temperature at 200°C. MS spectra were acquired under positive ionization mode and collected in the 50–2000 m/z range, both for MS and for MS/MS spectra. Bufotenine presence in tissues was evaluated by the detection of the precursor bufotenine ion (205.135* m/z*) and its daughter fragment of 160.064* m/z*. Instrument control, data acquisition, and data processing were performed with LabSolutions (LC-MS solution 3.60.361 version, Shimadzu Corp.).

### 2.6. Histology

The samples were preserved in 4% formaldehyde (made from paraformaldehyde) buffered in 0.1 M phosphate buffered saline (PBS), pH 7.2 [24] for 48 h, dehydrated in ethanol, and embedded in paraffin. Sections 4-6 *μ*m thick were obtained in a Leica RM2255 microtome with the use of disposable steel blades and were stained with hematoxylin and eosin (HE). Micrographs were taken with an Olympus BX51 light microscope equipped with a digital camera and Image-Pro Express software (Media Cybernetics).

### 2.7. Statistical Analysis

The statistical analyses were performed using two-way ANOVA followed by Bonferroni (multicomparisons) posttest. P value summary: there were no significant differences p > 0.05,  ^*∗*^ p ≤ 0.05,  ^*∗∗*^ p ≤ 0.01,  ^*∗∗∗*^ p ≤ 0.001.

## 3. Results

### 3.1. *In Vivo* Experiments

**Figure 1:**
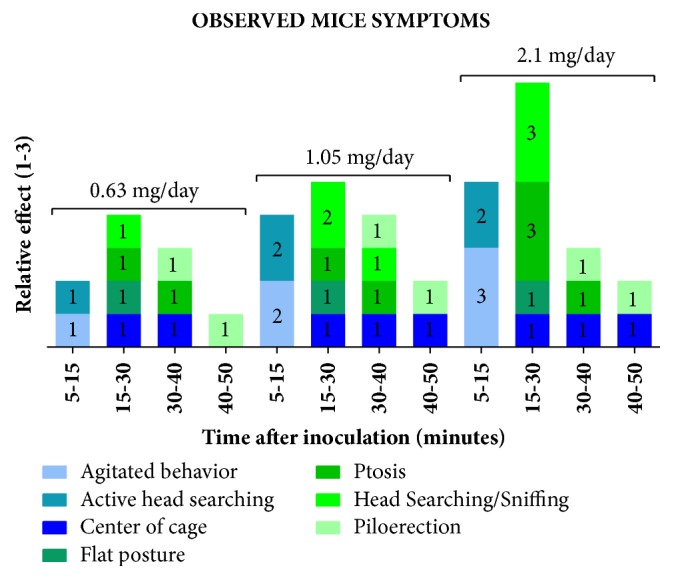
Observed symptoms and relative effect in mice after bufotenine inoculation.

Animals treated with bufotenine displayed different symptoms, which varied in intensity, during the first 50 minutes after the inoculation (Figure 1). The control group did not display any effect. In general, dose-dependent agitated behavior was observed few minutes after the injection, followed by ptosis, head searching, and sniffing. The parameters “remaining in the center of the cage” and “ptosis” were observed during the whole experiment, but were not dose-dependent.

**Figure 2:**
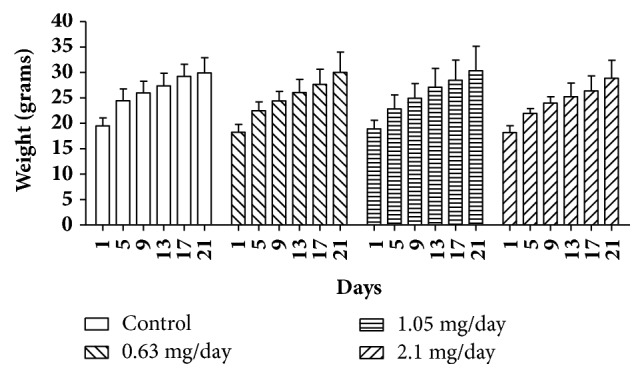
Average body weight of mice treated with NaCl 250 *μ*l/animal/day (control group) and mice treated with bufotenine 0.63, 1.05, and 2.1 mg in 250 *μ*l of NaCl/animal/day. Data are mean with SD, n = 10, two-way ANOVA followed by Bonferroni (multicomparisons) posttest. There were no significant differences compared with control (p > 0.05).

Treated animals were weighted on the first day of experiment and on days 5, 9, 13, 17, and 21 before the euthanasia, and body weight was compared. As seen in Figure 2, animals from all groups gained body weight, indicating that bufotenine did not cause any change in the mice general physiology. The difference among the groups was not statistically significant in any of the days when compared with the control or between the groups (p > 0.05). Comparing males and females, there was statistical difference (p < 0.05) only on day 5, in which females treated with 1.05 mg/day and males treated with 2.1 mg/day presented lower body weight, and on day 17, in which females treated with 2.1 mg/day showed lower mass values (Supplementary Figure 1). However, for all situations, body weight was recovered in the next measure.

### 3.2. Open Field Test

**Figure 3:**
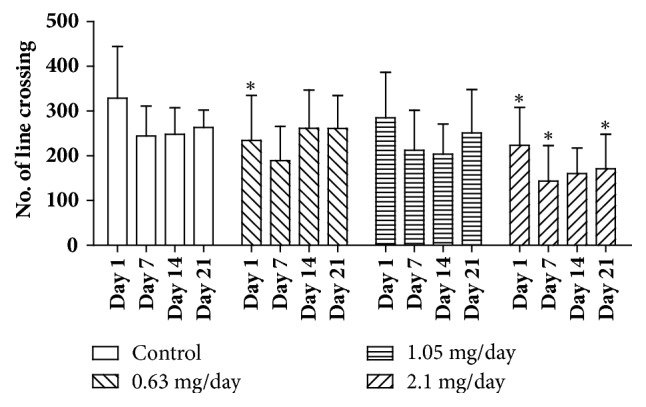
Number of line-crossing events on open field test of mice treated with NaCl 250 *μ*l/animal/day (control group) and mice treated with bufotenine 0.63, 1.05, and 2.1 mg in 250 *μ*l of NaCl/animal/day. The open field experiments were performed on days 1, 7, 14, and 21. Data are mean with SD, n = 10, two-way ANOVA followed by Bonferroni (multicomparisons) posttest. Significant differences compared with control are indicated with  ^*∗*^ (p < 0.05).

Figure 3 shows that the administration of 0.63 mg.mL^−1^ bufotenine caused a statistically significant decrease in the number of line-crossing events on day 1, when compared to the control group. On the other hand, the group treated with the higher dose (2.1 mg/day) decreased the number of line-crossing events in all days of treatment. We also performed data analyses on bufotenine open field effects discriminating male and female subjected. However, no apparent gender-effected could be perceived (Supplementary Figure 2).

**Figure 4:**
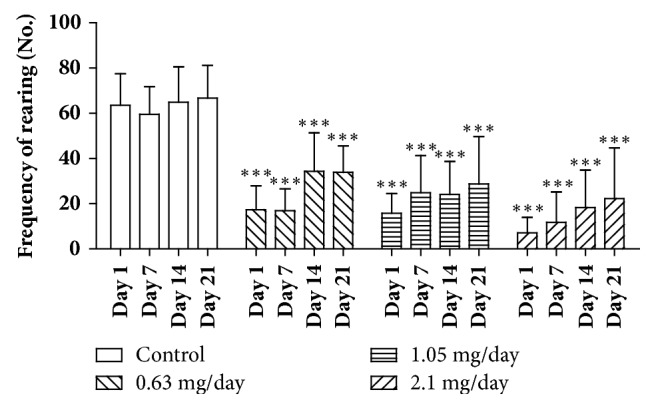
Frequency of rearing on open field test of mice treated with NaCl 250 *μ*l/animal/day (control group) and mice treated with bufotenine 0.63, 1.05, and 2.1 mg in 250 *μ*l of NaCl/animal/day. The open field experiments were performed on days 1, 7, 14, and 21. Data are mean with SD, n = 10, two-way ANOVA followed by Bonferroni (multicomparisons) posttest. Significant differences compared with control are indicated with  ^*∗∗∗*^ (p < 0.001).

**Figure 5:**
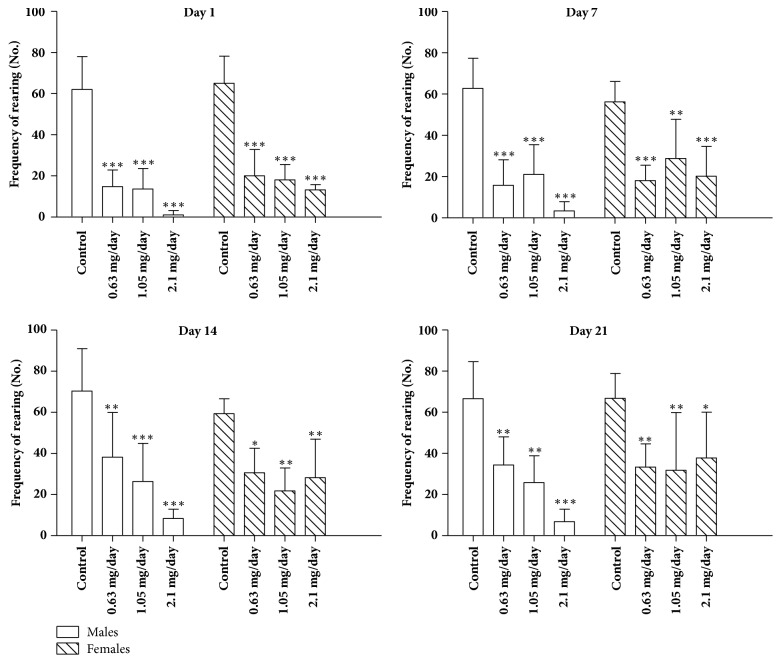
Frequency of rearing on open field test of males and females mice treated with NaCl 250 *μ*l/animal/day (control group) and males and females mice treated with bufotenine 0.63, 1.05, and 2.1 mg in 250 *μ*l of NaCl/animal/day. The open field experiments were performed on days 1, 7, 14, and 21. Data are mean with SD, n = 5, two-way ANOVA followed by Bonferroni (multicomparisons) posttest. Significant differences compared with control are indicated with  ^*∗*^ (p < 0.05),  ^*∗∗*^ (p < 0.01), and  ^*∗∗∗*^ (p < 0.001).

The number of rearing events, on the other hand, was significantly decreased (p < 0.001) in all bufotenine concentrations tested in any of the days of test (Figure 4), when compared with control. There was no significant difference among treated groups when compared to each other. Comparing males and females, in general, the males presented higher response to the treatment, mostly with the higher dose of 2.1 mg/day (Figure 5).

**Figure 6:**
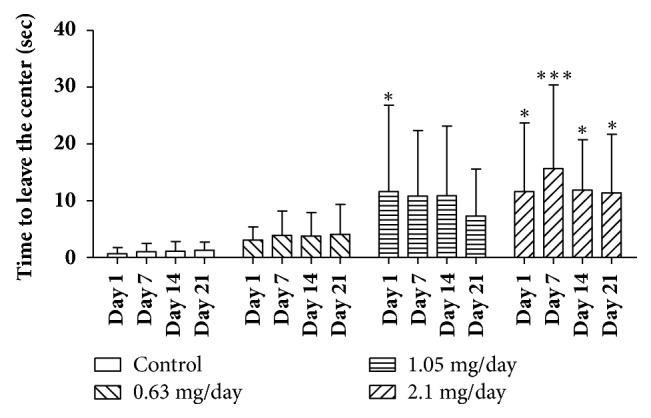
Time to leave the center (sec) on open field test of mice treated with NaCl 250 *μ*l/animal/day (control group) and mice treated with bufotenine 0.63, 1.05, and 2.1 mg in 250 *μ*l of NaCl/animal/day. The open field experiments were performed on days 1, 7, 14, and 21. Data are mean with SD, n = 10, two-way ANOVA followed by Bonferroni (multicomparisons) posttest. Significant differences compared with control are indicated with  ^*∗*^ (p < 0.05) and  ^*∗∗∗*^ (p < 0.001).

Bufotenine increased the time that animals spent to leave the center of the open field, mainly in the highest tested bufotenine concentration (Figure 6), from the first day of treatment. Comparing males and females, in general, the males seem to be more responsive (Supplementary Figure 3).

**Figure 7:**
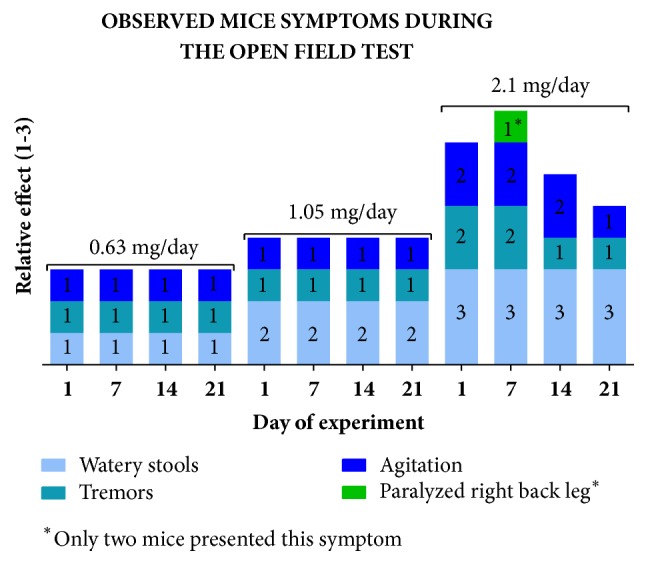
Observed symptoms and relative effect in mice after bufotenine inoculation during the open field test.

Although mice treated with bufotenine, in all concentrations, presented watery stools (see Figure 7), there were no significant differences in the frequency of defecation or urination (Supplementary Figures 4 and 5). Comparing males and females, there were also no significant differences (data not shown).

The open field tests also revealed symptoms that could not be evaluated on caged animals. Such symptoms are shown in Figure 7 and include watery stools, tremors, agitation, and limb paralysis. Moreover, despite the fact that we have not quantified this behavior, it was possible to observe that bufotenine treated mice tended to dwell in the center quadrants of the field, whereas control group animals wandered around peripheral quadrants.

### 3.3. Bufotenine Evaluation in Organs

For the analysis of the presence of bufotenine in mice organs, brain, liver, heart, kidney, lung, pancreas, and spleen were collected. Retention time and the MS and MS^2^ spectra were compared with the spectra of bufotenine standard (Supplementary Figure 6), which clearly shows the* m/z* of 205.135 and its fragmentation of 160.064* m/z* [25]. As shown in Table 2,* m/z* corresponding to bufotenine could be found in brain, heart, lung, and kidney but* m/z* corresponding to the alkaloid or its fragmentation was not detected in any of the organs of the control animals.

### 3.4. Histology

The animals treated with bufotenine showed hair loss on the inoculation area, that was more evident on mice treated with the highest concentration. After mice euthanasia, these areas (from both control and treated groups) were collected for histological analysis.

**Figure 8:**
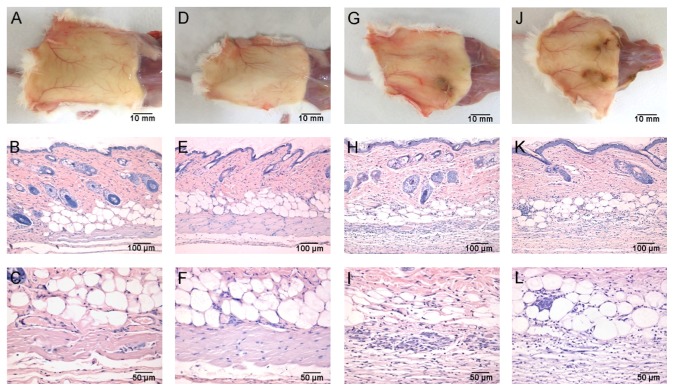
Analysis of the mice skin after treatment with bufotenine. Upper are the macroscopic internal images and lower are the histology from the injection site. (a), (b), and (c) = control; (d), (e), and (f) = 0.63 mg bufotenine; (g), (h), and (i) = 1.05 mg bufotenine; (j), (k), and (l) = 2.1 mg bufotenine.

As presented on Figure 8, the internal face of the skin from animals treated with 1.05 and 2.1 mg/day bufotenine had dark spots in the injected areas, resembling necrotic reaction. These areas, when analyzed by light microscopy, showed clear inflammatory process in the deep dermis identified by the extensive cell influx especially in the adipose layer and in the muscular layer. The muscle fibers were degraded, more markedly in the dose of 2.1 mg. The epidermis showed some spots of desquamation of the cornified layer, whereas the hair bulbs did not seem to be affected. On the other hand, no histological alterations were observed in the lower dose of 0.63 mg.

**Figure 9:**
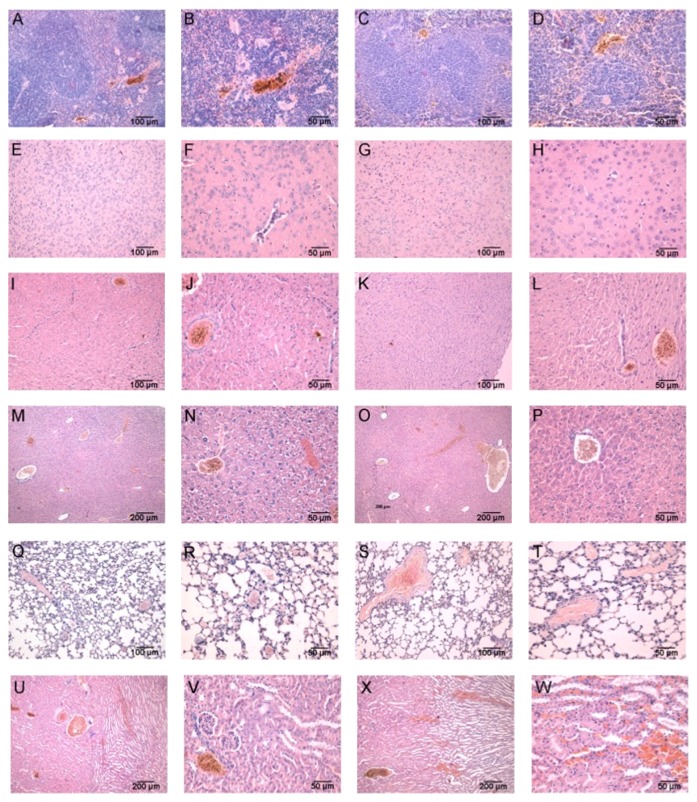
Histological analyses of organs from animals treated with bufotenine. (a) to (d) = spleen ((a) and (b) from control group and (c) and (d) from 2.1 mg bufotenine); (e) to (h) = brain ((e) and (f) from control group and (g) and (h) from 2.1 mg bufotenine); (i) to (l) = heart ((i) and (j) from control group and (k) and (l) from 2.1 mg bufotenine); (m) to (p) = liver ((m) and (n) from control group and (o) and (p) from 2.1 mg bufotenine); (q) to (t) = lung ((q) and (r) from control group and (s) and (t) from 2.1 mg bufotenine); (u) to (w) = kidney ((u) and (v) from control group and (x) and (w) from 2.1 mg bufotenine).

All the organs removed from the treated animals, when analyzed macroscopically, had the same aspect, compared to the control group (data not shown). In the same way, when histologically analyzed, the organs did not show alterations in any dose used, neither in their structure nor in terms of inflammatory response (see comparison between control and 2.1 mg/day doses in Figure 9 and comparison of all doses in Supplementary Figure 7).

## 4. Discussion

The term psychedelic, used to describe the effects of psychoactive drugs such as lysergic acid diethylamide (LSD), was coined in 1957 by the British psychiatrist Humphry Osmond [26]. These drugs belong to a group of others substances named as hallucinogens, that also includes deliriants and dissociatives drugs [27]. It is well established that psychedelic drugs behave as receptor 5-HT2A agonists or partial agonists [28–30]. Moreover, bufotenine and the hallucinogens LSD, 5-MeO-DMT, and psilocin have been reported to bind at both the 5-HT2A and 5-HT2C serotonin receptors [31–35].

Independently of bufotenine being able or not to cause hallucinogenic effects, in this article, we focused on the evaluation of the biological and toxicological effects of this alkaloid on mice and on its distribution in the organism.

Animals treated with bufotenine displayed different intensity of symptoms during the first 50 minutes after inoculation. Some of these symptoms were more intense in animals treated with higher doses, characterizing a concentration-response effect. After the tenth day of experiments, these signals seem to remain more lenient. The effects seem to be both peripheral and central, according to the symptoms observed and to the availability of serotonin receptor through the whole body [36].

The chosen model (open field test) allows the systematic assessment of specific rodent behaviors, such as novel environment exploration and locomotor activity, but serves as an initial screen for anxiety-related comportment [23].

In the open field test, increases in locomotion around the center and in the time spent in the center (or the time spent to leave it), without changes in the line-crossing events or in the frequency of rearing, are indicators of an anxiolytic-like effect. Anxiogenic-like effects, on the other hand, cause exactly the opposite, e.g., decreased central locomotion, decreased time spent at the center, and diminished line-crossing and rearing events.

Our results showed that bufotenine decreased the number of line-crossing events (at the highest dose, Figure 3) and also induced an evident decrease of rearing (Figure 4), behaviors that can be related to anxiogenic-like effects. The increase of the time spent in the center, the only indicator of anxiolytic-like effect, can be, on the other hand, associated with a possible sedative effect [23]. Sedation would corroborate the observations of paralyzed right back limbs observed in 20% of mice treated with the highest dose (Figure 7). Moreover, [37] tested the hallucinogen molecule DOI (1-(2,5-dimethoxy-4-iodophenyl)-2-aminopropane) on mice and, according to their results, the 5-HT2A and 5-HT2C receptors would arise contrasting effects on the locomotor activity (namely, an increase or a decrease in the activity, respectively). The fact that bufotenine does bind to either receptor [32–35] would explain the opposing observed effects.

Although mice treated with bufotenine showed some anxiogenic-like effects, there were no significant differences in the frequency of defecation and urination, in which increasing, according to [38], could be a measure of anxiety level in rodents. However, some authors have questioned the validity of these assessments, arguing that the frequency of defecation and urination could be related to signs of emotionality and not necessarily to levels of anxiety [39, 40]. Although watery stool was the most significant parameter affected in the open field test, it did not compromise the animals' health, as assessed by the body weight parameter (Figure 2).

One of the major debates regarding bufotenine effects is whether it does show hallucinogen activity. Even though it displays structural similarity to other hallucinogens like LSD and psilocin, it has low lipid solubility. As a consequence, some authors have suggested that bufotenine would not be able to cross the blood-brain barrier (BBB) [41–44]. However, [45] showed that, in rats, bufotenine was capable of penetrating the BBB being detected in different brain regions.

There are some structural similarities between bufotenine and others serotonergic hallucinogen alkaloids like psilocin, for example. Both molecules are hydroxylated at adjacent points on the indole ring system, pointing out to similar partition coefficients. Nevertheless, psilocin displays a partition coefficient of 3.30, whereas bufotenine figure is only 0.06 [41, 43]. Authors proposed that psilocin may form a pseudo-ring system, which would increase the permeability across the BBB, as well as a possible inhibition of monoamine oxidase, leading to oral efficacy [6, 46]. Moreover, the substitution of the methyl group of bufotenine, yielding 5-MeO-DMT, yields higher partition coefficient at the expense of a nonpolar molecule [43].

Fuller et al. [45] have demonstrated that, in rats, the 30 mg.kg^−1^ bufotenine dose (which corresponds to the initial dose in our experiments) was barely detectable in the lung and heart, and undetectable in the blood, brain, and liver after 8 hours of a single subcutaneous inoculation. Our experiments showed that bufotenine could be detected in brain, heart, lung, and kidney, even 24 hours after the last inoculation, suggesting that (i) the chronic administration may lead to the accumulation of this alkaloid in some organs; or (ii) bufotenine metabolization and/or excretion take longer than previously described. It is important to emphasize that Fuller et al. (1995) did not evaluate the presence of bufotenine in the kidney, impairing a thorough biodistribution comparison. Nevertheless, even if bufotenine does accumulate in certain organs, it does not cause structural damage and apparently does not indicate functional disturbances. Moreover, the lack of detection of the alkaloid in the liver, but its presence in the kidney, can indicate renal metabolism and elimination by urine [47].

Bufotenine toxicity in rodents has been estimated to be 200 ~ 300 mg.kg^−1^. At higher doses, bufotenine increases the respiratory rate and induces nausea, vomiting, and other gastrointestinal effects [48, 49]. Although the presence of watery stools reported here could be related to gastrointestinal disturbance, the other reported effects were not observed in this study, corroborating our observation that bufotenine is active against rabies (manuscript in preparation) at doses below being toxic.

In conclusion, we report that 0.63 mg/day dose of bufotenine, which is the effective dose against rabies infection, did not cause significant effects on the animals' physiology and only a few (not significant as well) effects on the CNS. Moreover, the higher employed doses (1.05 and 2.1 mg/day) were not toxic as well, according to the homogenous body weight gain, negative histopathology alterations, and mild behavioral effects. Taking into account the fact that rabies is an incurable disease, bufotenine might be considered as a drug prototype, in spite of the few anxiogenic observed effects.

## Figures and Tables

**Table 1:** Bufotenine structure and related bioactive molecules.

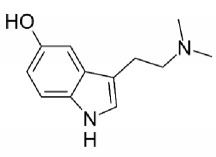	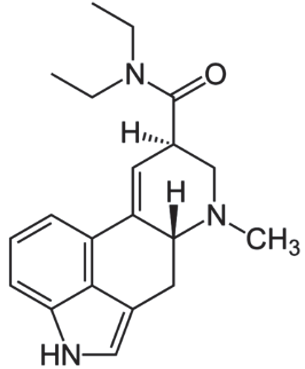	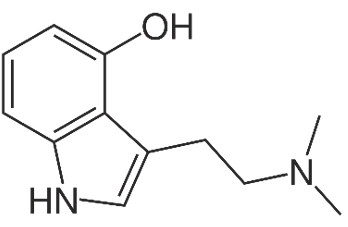

Bufotenine	LSD	Psilocin

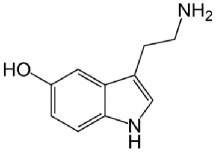	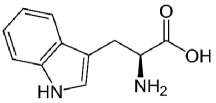	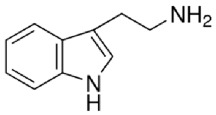

Serotonin	Tryptophan	Tryptamine

**Table 2:** Detection of bufotenine in mice organs.

		Bufotenine concentration		
	Control	0.63 mg/day	1.05 mg/day	2.1 mg/day
Spleen	-	-	-	-
Brain	-	-	-	+
Heart	-	+	+	+
Liver	-	-	-	-
Lung	-	-	+	+
Kidney	-	+	+	+

-: not detectable; +: detectable.

## Data Availability

The data used to support the findings of this study are available from the corresponding author upon request.

## Additional Points

*Highlights*. Bufotenine, an alkaloid with antirabies activity, caused no significant toxicity in mice in the therapeutic dose. The alkaloid caused histological alteration only in the site injection with high dose and not in organs and tissues. Mild effects on CNS could be observed with high doses of bufotenine. Bufotenine could be found in the heart, lung, kidney, and brain.

## Supplementary Materials

Supplementary Materials Supplementary Figure 1: average body weight of males and females mice treated with NaCl 250 *μ*l/animal/day (control group) and males and females mice treated with bufotenine 0.63, 1.05, and 2.1 mg in 250 *μ*l of NaCl/animal/day. Data are mean with SD, n = 5, two-way ANOVA followed by Bonferroni (multicomparisons) posttest. Significant differences compared with control are indicated with  ^*∗*^ (p < 0.05). Supplementary Figure 2: number of line-crossing events on open field test of males and females mice treated with NaCl 250 *μ*l/animal/day (control group) and males and females mice treated with bufotenine 0.63, 1.05, and 2.1 mg in 250 *μ*l of NaCl/animal/day. The open field experiments were performed on days 1, 7, 14, and 21. Data are mean with SD, n = 5, two-way ANOVA followed by Bonferroni (multicomparisons) posttest. Significant differences compared with control are indicated with  ^*∗*^ (p < 0.05) and  ^*∗∗*^ (p < 0.01). Supplementary Figure 3: time to leave the center (sec) on Open Field test of males and females mice treated with NaCl 250 *μ*l/animal/day (control group) and mice treated with bufotenine 0.63, 1.05, and 2.1 mg in 250 *μ*l of NaCl/animal/day. The open field experiments were performed on days 1, 7, 14, and 21. Data are mean with SD, n = 5, two-way ANOVA followed by Bonferroni (multicomparisons) posttest. Significant differences compared with control are indicated with  ^*∗*^ (p < 0.05),  ^*∗∗*^ (p < 0.01), and  ^*∗∗∗*^ (p < 0.001). Supplementary Figure 4: frequency of defecation on open field test of mice treated with NaCl 250 *μ*l/animal/day (control group) and mice treated with bufotenine 0.63, 1.05, and 2.1 mg in 250 *μ*l of NaCl/animal/day. The open field experiments were performed on days 1, 7, 14, and 21. Data are mean with SD, n = 5, two-way ANOVA followed by Bonferroni (multicomparisons) posttest. There were no significant differences compared with control (p > 0.05). Supplementary Figure 5: frequency of urination on open field test of mice treated with NaCl 250 *μ*l/animal/day (control group) and mice treated with bufotenine 0.63, 1.05, and 2.1 mg in 250 *μ*l of NaCl/animal/day. The open field experiments were performed on days 1, 7, 14, and 21. Data are mean with SD, n = 5, two-way ANOVA followed by Bonferroni (multicomparisons) posttest. There were no significant differences compared with control (p > 0.05). Supplementary Figure 6: bufotenine standard MS and MS^2^ spectra showing the* m/z* of 205.135 (A) and its fragmentation of 160.064* m/z *(B). Supplementary Figure 7: histological analyses of organs (spleen, brain, heart, liver, lung, and kidney) after treatment with different doses of bufotenine (0.63, 1.05, and 2;1 mg).
