# Supplementary material for: Biological Effects and Biodistribution of Bufotenine on Mice
Source: Biomed Res Int. 2018 May 31;2018:1032638. doi: 10.1155/2018/1032638 (PMC6000854; doi:10.1155/2018/1032638)
Supplement: Supplementary Materials — Supplementary Figure 1: average body weight of males and females mice treated with NaCl 250 μl/animal/day (control group) and males and females mice treated with bufotenine 0.63, 1.05, and 2.1 mg in 250 μl of NaCl/animal/day. Data are mean with SD, n = 5, two-way ANOVA followed by Bonferroni (multicomparisons) posttest. Significant differences compared with control are indicated with ∗ (p < 0.05). Supplementary Figure 2: number of line-crossing events on open field test of males and females mice treated with NaCl 250 μl/animal/day (control group) and males and females mice treated with bufotenine 0.63, 1.05, and 2.1 mg in 250 μl of NaCl/animal/day. The open field experiments were performed on days 1, 7, 14, and 21. Data are mean with SD, n = 5, two-way ANOVA followed by Bonferroni (multicomparisons) posttest. Significant differences compared with control are indicated with ∗ (p < 0.05) and ∗∗ (p < 0.01). Supplementary Figure 3: time to leave the center (sec) on Open Field test of males and females mice treated with NaCl 250 μl/animal/day (control group) and mice treated with bufotenine 0.63, 1.05, and 2.1 mg in 250 μl of NaCl/animal/day. The open field experiments were performed on days 1, 7, 14, and 21. Data are mean with SD, n = 5, two-way ANOVA followed by Bonferroni (multicomparisons) posttest. Significant differences compared with control are indicated with ∗ (p < 0.05), ∗∗ (p < 0.01), and ∗∗∗ (p < 0.001). Supplementary Figure 4: frequency of defecation on open field test of mice treated with NaCl 250 μl/animal/day (control group) and mice treated with bufotenine 0.63, 1.05, and 2.1 mg in 250 μl of NaCl/animal/day. The open field experiments were performed on days 1, 7, 14, and 21. Data are mean with SD, n = 5, two-way ANOVA followed by Bonferroni (multicomparisons) posttest. There were no significant differences compared with control (p > 0.05). Supplementary Figure 5: frequency of urination on open field test of mice treated with NaCl 250 μl/animal/day (contr [file 1032638.f1.docx]

**Graphical abstract**


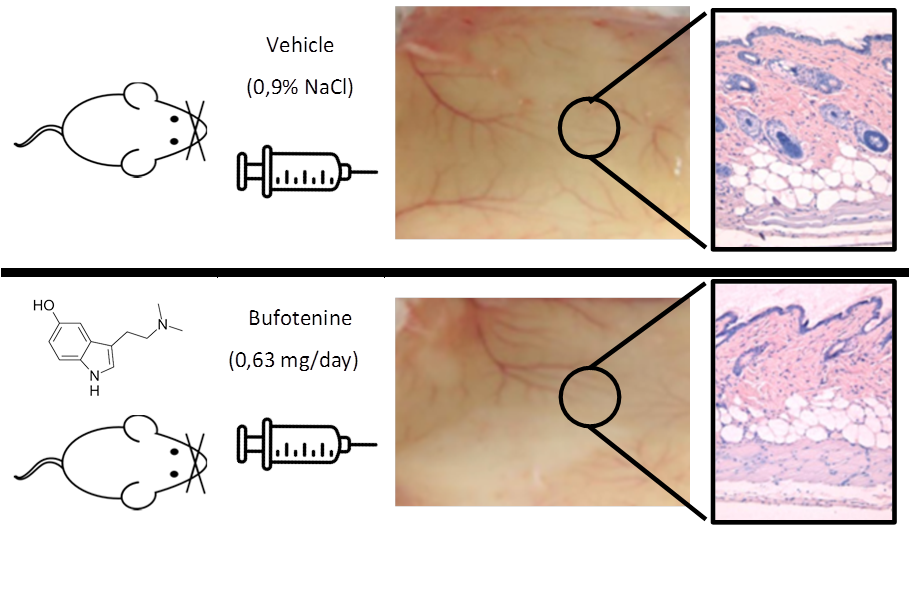

Supplementary Fig. 1

Supplementary Fig. 2

Supplementary Fig. 3

Supplementary Fig. 4

Supplementary Fig. 5

Supplementary Fig. 6


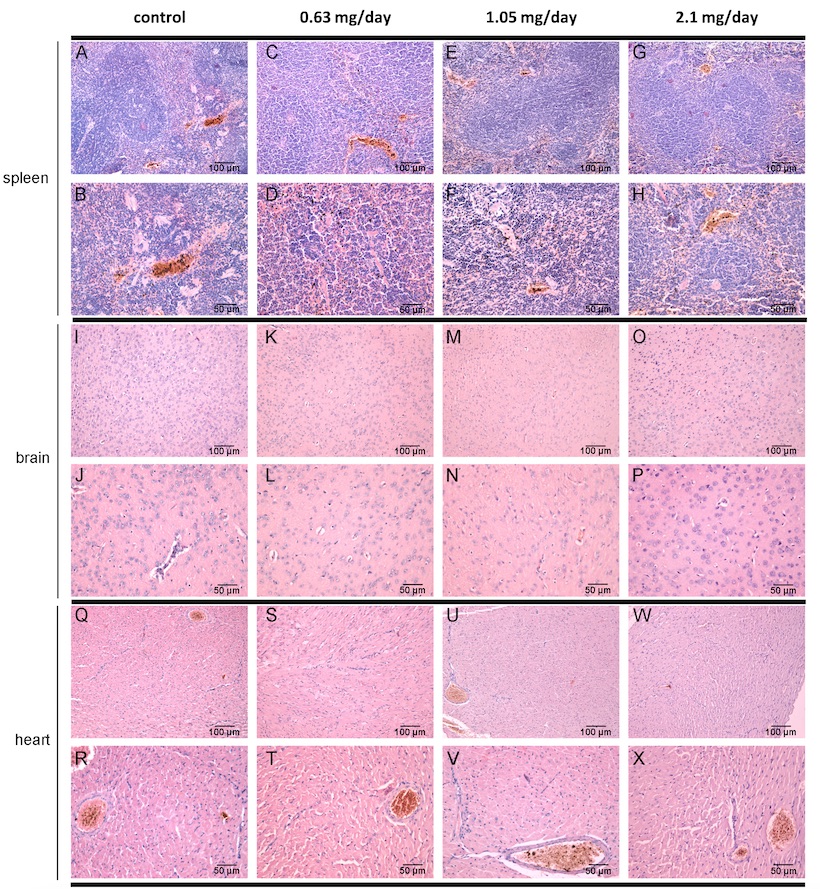

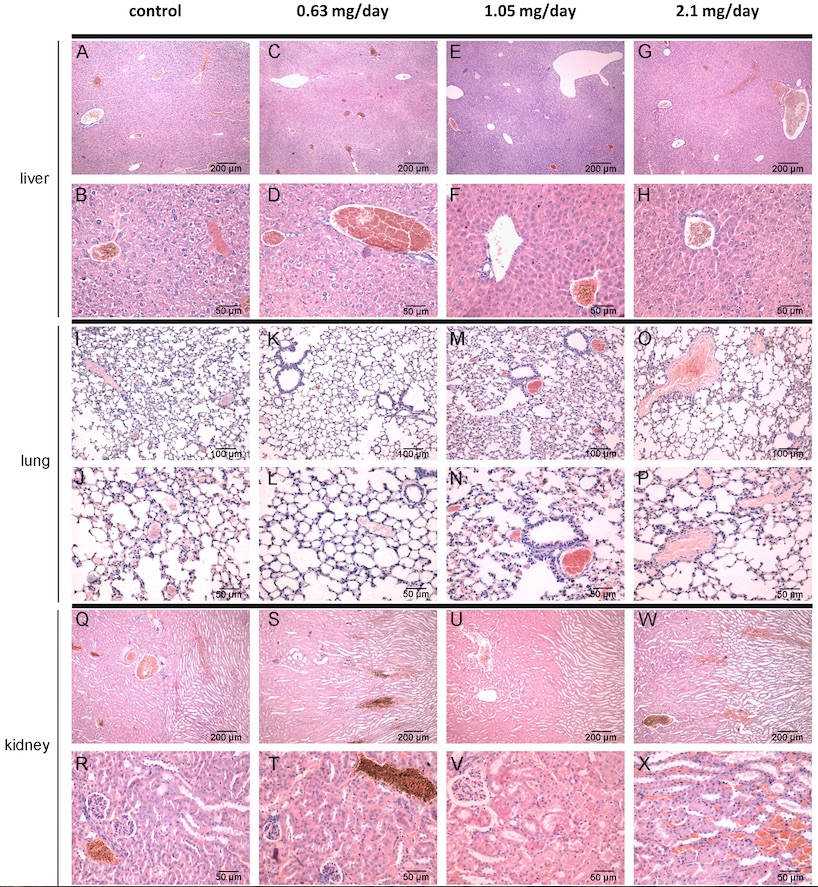


Supplementary Fig. 7
